# Prognostic and Clinicopathological Significance of Survivin Expression in Renal Cell Carcinoma: A Systematic Review and Meta-Analysis

**DOI:** 10.1038/srep29794

**Published:** 2016-07-14

**Authors:** Yongpeng Xie, Xin Ma, Liangyou Gu, Hongzhao Li, Luyao Chen, Xintao Li, Yu Gao, Yang Fan, Yu Zhang, Yuanxin Yao, Xu Zhang

**Affiliations:** 1 State Key Laboratory of Kidney Diseases, Department of Urology, Chinese PLA Medical School, Chinese PLA General Hospital, Beijing, People’s Republic of China; 2 Medical School, Nankai University, Tianjin, People’s Republic of China

## Abstract

Previous studies have elevated the prognostic value of survivin in renal cell carcinoma (RCC). To increase statistical power and improve translation, we systematically searched PubMed, Web of Science, and Embase to identify relevant studies until December 2015 and conducted a standard meta-analysis. Based on the inclusion and exclusion criteria, a total of 12 studies, including 2051 patients, were eligible for further analysis. Results showed that high survivin expression in RCC was associated with poor OS (HR = 2.84, 95% CI 1.68–4.79), CSS (HR = 2.36, 95% CI 1.41–3.95), and PFS (HR = 2.20, 95% CI 1.58–3.08). Survivin expression was also correlated with TNM stage (RR = 2.75, 95% CI 2.21–3.44), pathological T stage (RR = 2.19, 95% CI 1.75–2.75), lymph node metastasis (RR = 2.28, 95% CI 1.61–3.25), distant metastasis (RR = 1.56, 95% CI 1.16–2.08), Fuhrman grade (RR = 2.81, 95% CI 2.29–3.45), tumor size (RR = 1.49, 95% CI 1.24–1.78). Our study suggested that survivin was a prognostic marker in RCC. High survivin expression was correlated with poor prognosis and more advanced clinicopathological features, and it could serve as a biomarker for disease management.

Renal cell carcinoma (RCC) accounts for 3% of all human malignancies and is the third most prevalent genitourinary cancers^1^. RCC is highly aggressive. Approximately 30% of patients have metastases at first diagnosis, and another 20% of RCC patients with clinically localized disease will develop metastasis even after curative nephrectomy^2^. Most metastatic RCCs still eventually cause death in spite of the application of targeted therapy^3^. In this regard, prediction models identifying patients with poor prognosis, who may benefit from early systematic therapy, are greatly needed. To date, the tumor, node, and metastasis (TNM) staging system is a widely used RCC prognostic predictor. However, such classic clinical and pathological factors fail to address the inherent biological heterogeneity of RCC^4^. Therefore, novel biomarkers that can stratify patients with poor prognosis of RCC are required to guide clinical decisions precisely.

Survivin is a member of the inhibitor of apoptosis protein family and is usually present in embryonic tissues^5^. Survivin plays a role in cell cycle regulation, inhibition of apoptosis, angiogenesis, and other biological effects^6^. Intriguingly, survivin is barely detectable in most normal adult tissues but overexpressed in many cancers, including RCC^7^. With further understanding of the molecular mechanisms of RCC, numerous studies focusing on survivin have been conducted in the fields of outcome prediction and potential therapeutic targets. To obtain a more precise evaluation of the prognostic and clinicopathological value of survivin expression in RCC, we conducted a systematic review and meta-analysis to evaluate the prognostic value of survivin quantitatively and explore the associations of survivin with the clinicopathological features of RCC.

## Results

### Search Results

**Figure 1:**
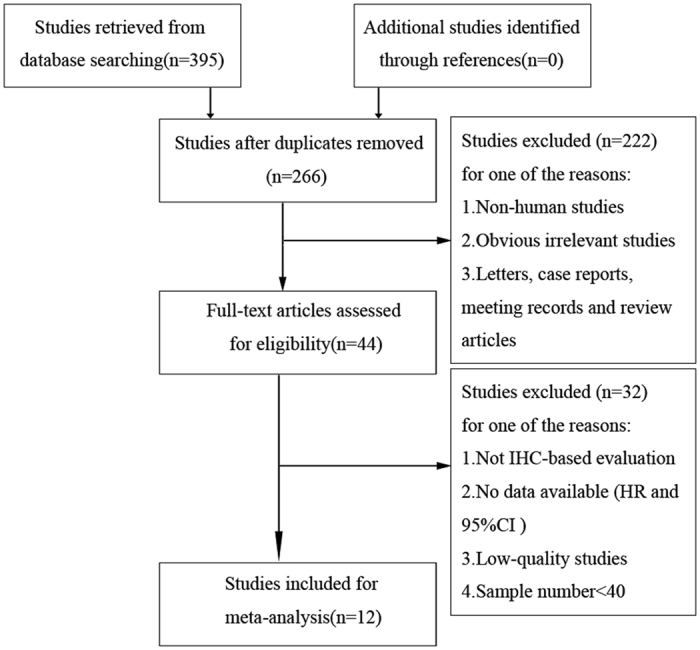


A total of 395 articles were retrieved from the primary literature search. A total of 129 duplicate reports were excluded. After screening the titles and abstracts, 222 articles were excluded for reasons such as non-human studies, letters, case reports, reviews, and other obvious irrelevant studies. The remaining articles were viewed in full text. To avoid the heterogeneity caused by the detection method, studies without IHC evaluation were excluded, and the remaining articles were further excluded for several reasons, such as no data available (HR and 95% CI), low-quality studies^8^, samples fewer than 40, and duplicate publication. Finally, only 12 articles with 2051 patients satisfied the criteria for meta-analysis^9^,^10^,^11^,^12^,^13^,^14^,^15^,^16^,^17^,^18^,^19^,^20^. A flowchart of the study selection process is shown in Fig. 1.

### Characteristics of Studies

The detailed data of the 12 studies are summarized in Table 1. All of the included studies were published recently (2005–2015). Patients in these studies were all diagnosed with RCC with different tumor types and received radical or partial nephrectomy. Five studies originated from the United States, four from China, one from Germany, one from Italy, and one from Korea. Among the studies, four studies were carried out to analyze OS, seven studies were conducted to investigate CSS, and four studies reported PFS. Various clinicopathological data were reported in seven studies (TNM stage in five studies, pathological T stage in five studies, lymph node metastasis in six studies, distant metastasis in four studies, Fuhrman grade in six studies, tumor size in four studies). All studies applied immunohistochemical staining to investigate survivin expression. The cutoff values of positive survivin expression varied among different studies, so we classified all the cases according to their original studies (negative or positive staining).

### Meta-Analysis

**Figure 2:**
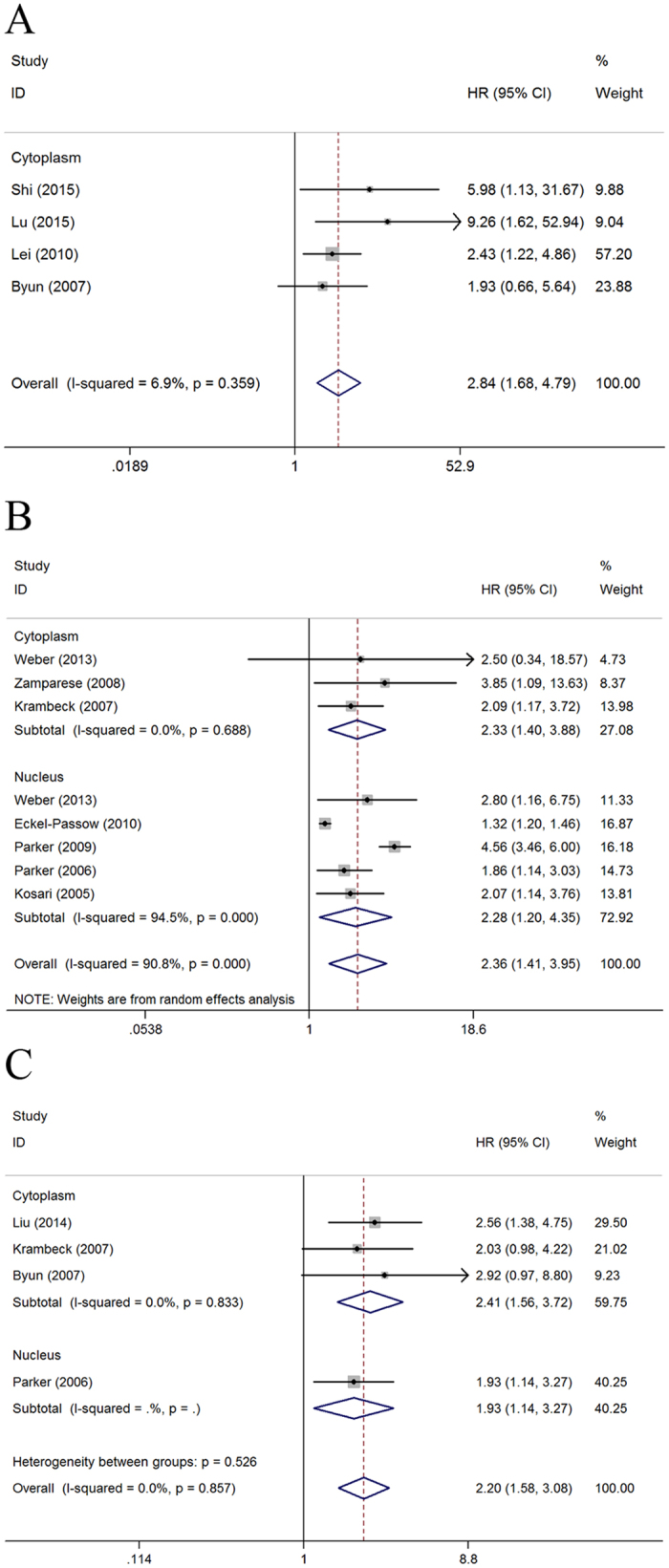
Forest plots of studies evaluating the association of survivin expression and prognostic outcomes of RCC patients and subgroup analysis in terms of different subcellular localization of survivin expression. (**A**) effect of survivin overexpression on OS, (**B**) CSS, and (**C**) PFS. HR: hazard ratio; CI: confidence interval; OS: overall survival; CSS: cancer-specific survival; PFS: progression-free survival; RCC: renal cell carcinoma. HR > 1 implies unfavorable prognosis for patients with high survivin expression.

The results showed that high survivin expression in RCC was associated with poor OS (a fixed-effect model, HR = 2.84; 95% CI: 1.68–4.79; p < 0.001; *I*^*2*^ = 6.9%, p = 0.359; Fig. 2A), CSS (a random-effect model, HR = 2.36; 95% CI: 1.41–3.95; p < 0.001; *I*^*2*^ = 90.8%, p < 0.001; Fig. 2B), and PFS (a fixed-effect model, HR = 2.20; 95% CI: 1.58–3.08; p < 0.001; *I*^*2*^ = 0.0%, p = 0.857; Fig. 2C). Furthermore, subgroup analysis stratified by ethnicity, expression location, extent of tumor at time of diagnosis, histopathological subtype and cutoff of staining were also performed. With regard to ethnicity, high survivin expression was associated with poor OS (HR, 2.84; 95% CI: 1.68–4.79; p < 0.001) and PFS (HR, 2.64; 95% CI: 1.54–4.53; p < 0.001) in Asian patients; with poor CSS (HR, 2.36; 95% CI: 1.41–3.95; p = 0.001) and PFS (HR, 1.96; 95% CI: 1.28–3.01; p = 0.002) in Caucasian patients (Table 2). For expression location, high cytoplasmic expression of survivin was associated with poor OS (HR, 2.84; 95% CI: 1.68–4.79; p < 0.001), CSS (HR, 2.33; 95% CI: 1.40–3.88; p = 0.001), and PFS (HR, 2.41; 95% CI: 1.56–3.72; p < 0.001). High nuclear expression of survivin was associated with poor CSS (HR, 2.28; 95% CI: 1.20–4.35; p = 0.012) and PFS (HR, 1.93; 95% CI: 1.14–3.27; p = 0.015). Regarding extent of tumor, high survivin expression was correlated with poor OS (HR, 2.84; 95% CI: 1.68–4.79; p < 0.001), CSS (HR, 2.31; 95% CI: 1.30–4.10; p = 0.004), and PFS (HR, 2.20; 95% CI: 1.58–3.08; p < 0.001) for all stages of RCC; with poor CSS (HR, 2.75; 95% CI: 1.23–6.15; p = 0.014) for localized RCC. For histopathological subtype, high survivin expression was correlated with poor OS (HR, 7.37; 95% CI: 2.21–24.58; p = 0.001), CSS (HR, 2.36; 95% CI: 1.41–3.95; p = 0.001), and PFS (HR, 2.14; 95% CI: 1.51–3.04; p < 0.001) for ccRCC. In the cutoff of staining subgroup analysis, high survivin expression was correlated with poor OS (HR, 5.98; 95% CI: 1.13–31.67; p = 0.036), CSS (HR, 1.95; 95% CI: 1.35–2.83; p < 0.001), and PFS (HR, 2.14; 95% CI: 1.51–3.04; p < 0.001) when the cutoff value was less than 10%. Studies with cutoff value over 10% showed that high survivin expression was related to poor OS (HR, 2.61; 95% CI: 1.51–4.54; p = 0.001) and poor CSS (HR, 3.03; 95% CI: 1.54–5.98; p = 0.001) but not to PFS (HR, 2.92; 95% CI: 0.97–8.80; p = 0.057).

In the comprehensive analyses of the role of survivin expression in RCC as a biomarker, we investigated the association of high survivin expression and clinicopathological characteristics. As reported in Table 3, high survivin expression was significantly associated with TNM stage (III/IV vs. I/II: RR, 2.75; 95% CI: 2.21–3.44; p < 0.001), pathological T stage (T3/T4 vs. T1/T2: RR, 2.19; 95% CI: 1.75–2.75; p < 0.001), lymph node metastasis (yes vs. no: RR, 2.28; 95% CI: 1.61–3.25; p < 0.001), distant metastasis (yes vs. no: RR, 1.56; 95% CI: 1.16–2.08; p = 0.003), Fuhrman grade (III/IV vs. I/II: RR, 2.81; 95% CI: 2.29–3.45; p < 0.001), and tumor size (>7 vs. ≤7: RR, 1.49; 95% CI: 1.24–1.78; p < 0.001). Some interstudy significant heterogeneity was observed in distant metastasis and tumor size, but analysis on other parameters did not exhibit significant heterogeneity.

### Sensitivity Analyses

To validate the reliability of our results and investigate the source of significant heterogeneity, sensitivity analysis was performed. Sensitivity analyses showed that the pooled HR was not significantly influenced after omitting any single study for the effect of survivin expression on CSS and revealed that the study^15^ was the source of statistical heterogeneity (Table 4). When this study was deleted, no significant heterogeneity was observed in the remaining studies (*I*^*2*^ = 47%, p = 0.079) and pooled HR was 1.39 (a fixed-effect model, 95% CI: 1.27–1.53; p < 0.001) or 1.84 (a random-effect model, 95% CI: 1.37–2.47; p < 0.001). No significant heterogeneity was detected in either OS or PFS; hence, we did not conduct sensitivity analysis.

### Publication Bias

**Figure 3:**
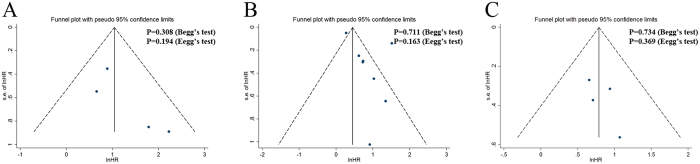
Funnel plots and Begg’s and Egger’s tests for the evaluation of potential publication bias. (**A**) Overall survival; (**B**) Cancer-specific survival; (**C**) Progression-free survival.

In the present meta-analysis, we introduced Begg’s and Egger’s tests, as well as funnel plots, to assess publication bias. As presented in Fig. 3, the funnel plots revealed that the included studies had no evident asymmetry. Further, the results from Begg’s test (P value) and Egger’s test (intercept with corresponding 95% CI, P value) for the included studies evaluating the survival outcomes were P_Begg’s_ = 0.308, intercept 1.97 with 95% CI −2.43 to 6.36, P_Egger’s_ = 0.194 (OS); P_Begg’s_ = 0.711, intercept 2.27 with 95% CI −1.22 to 5.76, P_Egger’s_ = 0.163 (CSS); P_Begg’s_ = 0.734, intercept 1.13 with 95% CI −3.11 to 5.38, P_Egger’s_ = 0.369 (PFS), respectively. Therefore, the aforementioned evidences suggested significant publication bias did not exist in our meta-analysis.

## Discussion

High expression of survivin is observed in many malignancies, but it is barely detectable in most normal adult tissues; thus, survivin is an attractive prognostic prediction marker and potential therapeutic target for several cancer types^21^,^22^,^23^,^24^. The new paper by Gulati *et al.* validated survivin gene expression is adverse implications in the prognosis of RCC^25^. To increase statistical power and improve translation, we conducted a meta-analysis to determine a pooled conclusion and provide evidence on the correlation.

In the current study, we focused exclusively on validating survivin IHC expression and evaluated the prognostic values of survivin IHC expression in RCC. We concluded that high survivin expression predicted poor prognosis in RCC patients. In particular, RCC patients with high survivin expression had shorter OS, CSS, and PFS. Subgroup analysis revealed that high survivin expression was significantly associated with poor OS and CSS, regardless of ethnicity, subcellular localizations, extent of tumor, histopathological subtype, and staining cutoff. Among patients with staining cutoff ≥10%, no significant association was identified between high survivin expression and poor PFS, even though patients with high survivin expression presented a relatively unfavorable PFS. The absence of a significant association was possibly due to the relatively limited studies in the subgroups. The reasons why high survivin expression may be of prognostic relevance in patients suffering from RCC remain speculative. Survivin is an anti-apoptotic protein that has been associated with cellular apoptosis inhibition function through preferential blocking of mitochondrial-dependent apoptosis by targeting caspase 9 and second mitochondria-derived activator of caspases/direct inhibitory apoptotic protein-binding protein with a low isoelectric point. Moreover, survivin presents a mitosis-regulated pattern of expression during the G2/M phase of the cell cycle. In addition to the widely accepted apoptosis inhibition function, survivin also plays a critical role in mitosis and microtubule stability^26^,^27^. Abnormal inhibition of apoptosis and cell division during cellular homeostasis is a critical process for the development and progression of RCC.

Consistent with previous reports, our results also suggested that RCC patients with high survivin expression were likely to have a higher TNM stage, pathological T stage, positive lymph nodes, distant metastasis, a higher Fuhrman grade, and a larger tumor size. Currently, survivin is believed to be related to angiogenesis by interacting with vascular endothelial growth factor, angiopoietin, and basic fibroblast growth factor. In addition, survivin is associated with metastatic behavior by activating various signaling pathways^28^. These mechanisms can explain the association between high survivin expression and clinicopathological characteristics in RCC patients.

This study is comprehensive analysis on the effect of survivin on prognostic and clinicopathological significance in patients with RCC, but several limitations should be pointed out. First, although all included studies measured survivin expression via IHC, the criteria to determine the positive or negative expression of survivin were inconsistent in different studies, which may generate heterogeneity of the overall results. Thus, a more standard cutoff value should be defined in the future. Second, marked heterogeneity of studies was observed in CSS analysis. The heterogeneity of CSS analysis was probably caused by differences in factors such as the patients’ characteristics (ethnicity, nationality, gender, age, and tumor stage and grade), variation of cut-off values for survivin expression, and different duration of follow-up. Third, in evaluating associations between survivin expression and clinicopathological characteristics of RCC, some studies lacked complete data, which may cause heterogeneity and contribute to the low reliability of the results. Furthermore, positive results were more likely to be published in most studies, whereas studies with negative results were often rejected or less assessable, which could lead to publication bias^29^, although this bias was not detected in the current analysis.

In conclusion, despite the abovementioned limitations, our meta-analysis suggested the prognostic and clinicopathological values of survivin expression in RCC. High survivin expression was correlated with poor prognosis and more advanced clinicopathological features, which may potentially serve as risk stratification markers and even therapeutic targets in RCC. However, more multicenter prospective studies with standardized methods and long-term follow up are needed to verify our results.

## Methods

### Search Strategy

This meta-analysis followed the guidelines of Preferred Reporting Items for Systematic Reviews and Meta-Analyses (PRISMA)^30^.

A systematic literature search was performed in the electronic databases PubMed, Web of Science, and Embase on 31 December 2015 using the following search strategy: (“survivin” or “BIRC5” or “baculoviral inhibitor of apoptosis repeat-containing 5”) and (“carcinoma” or “neoplasm” or “tumor” or “cancer” or “malignancy”) and (“kidney” or “renal”) and (“prognosis” or “prognostic” or “survival” or “outcome” or “mortality”). Furthermore, we manually searched the reference lists of relevant literature.

### Selection Criteria

Studies were included based on the following criteria: (1) the association of survivin with prognosis significance in RCC should be described; (2) studies detected survivin protein expression by immunohistochemistry(IHC); and (3) studies reported survival outcomes [overall survival (OS), cancer-specific survival (CSS), or progression-free survival (PFS)] with hazard ratio (HR) and 95% confidence interval (CI). Exclusion criteria were as follows: (1) non-English papers; (2) case reports, letters, commentaries, meeting records, or review articles; (3) sample number fewer than 40 patients; (4) the study focused on animal models or cancer cells; (5) the study did not analyze survivin protein expression, clinical features, and survival outcome; (6) the study lacked sufficient data for obtaining HR and 95% CI. All evaluations were independently performed by three individual researchers to ensure the accurate inclusion of studies. For duplicate studies, we only retrieved the most informative and recently studied one for further analyses.

Because this study was based on published literature, ethical approval from ethics committees was not needed.

### Data Extraction

Three investigators independently extracted data from eligible studies using a predefined form. Discrepancies in data extraction were resolved by discussion. The following data were extracted: surname of the first author, publication year, origin of the studied population, study design, extent of tumor, histopathological subtype, sample size, patient’s age, location of survivin expression, cutoff value, follow-up time, and effect estimates, namely, HR of survivin expression for OS, CSS, or PFS, as well as their 95% CI (Table 1). If the HR and 95% CI were not directly available, we calculated HRs and their 95% CI based on the methods reported by Tierney *et al.*^31^.

### Quality Assessment

The quality of the included studies was evaluated using the Newcastle–Ottawa scale, which was recommended by the Cochrane Non-Randomized Studies Methods Working Group^8^. Each study can be assessed by eight methodology items with a score ranging from 0 to 9. High scores indicated high quality, and we considered studies with scores of 6 or more as high quality for the meta-analysis.

### Statistical Analysis

Pooled HR and risk ratio (RR) with 95% CI were used to evaluate the association of survivin expression with RCC prognosis and clinicopathological characteristics, respectively. An observed HR > 1 implied worse prognosis for patients with high survivin expression. An observed RR > 1 implied more advanced clinicopathological characteristics for the group of high survivin expression. A heterogeneity test of pooled HR and RR was conducted using Cochran’s Q test and Higgins I-squared statistic. *I*^*2*^ values >50% indicated heterogeneity among studies^32^. When heterogeneity was observed (*I*^*2*^ > 50%), a random-effect model was used; otherwise, a fixed-effect model was used. For additional analyses, meta-analyses were subgrouped based on ethnicity, subcellular localizations, extent of tumor, histopathological subtype, and staining cutoff. For the investigation of heterogeneous studies, we also conducted sensitivity analysis to evaluate the influence of individual studies on the robustness of pooled results. Publication bias was assessed by funnel plot visual inspection and statistically evaluated by Begg’s^33^ and Egger’s tests^34^. All statistical analyses were performed using Stata 12.0 software (StatCorp, College Station, TX, USA) and p < 0.05 was considered statistically significant.

## Additional Information

**How to cite this article**: Xie, Y. *et al.* Prognostic and Clinicopathological Significance of Survivin Expression in Renal Cell Carcinoma: A Systematic Review and Meta-Analysis. *Sci. Rep.* **6**, 29794; doi: 10.1038/srep29794 (2016).

## Figures and Tables

**Table 1:** Characteristics of eligible studies in the meta-analysis.

Study	Country	Study design	Extent of tumor^a^	Histopathological subtype	Case number	Age (years)	Expression Location	Positive staining	follow-up (months)	Survival analysis	Quality score*
Kosari^12^	USA	Cohort study	all-stage	ccRCC	183	NA	N	IHC score^c^ ≥2	>28.8	CSS	7
Parker^11^	USA	Cohort study	all-stage	ccRCC	312	157/155(≥65 y/<65 y)^b^	N	≥2%	>26.4	CSS, PFS	8
Byun^10^	Korea	Cohort study	all-stage	ccRCC + non-ccRCC	85	Mean 53.2	C	≥10%	45	OS, PFS	7
Krambeck^13^	USA	Cohort study	all-stage	ccRCC	228	113/115(≥65 y/<65 y)	C	≥2%	>25.2	CSS, PFS	8
Zamparese^14^	Italy	Cohort study	all-stage	ccRCC	49	Mean 62	C	≥25%	47.1	CSS	6
Parker^15^	USA	Cohort study	all-stage	ccRCC	634	312/322(≥65 y/<65 y)	N	≥15 positive cells/mm^2^	>25.2	CSS	7
Eckel-Passow^16^	USA	Cohort study	all-stage	ccRCC	100	NA	N	NA	>19.2	CSS	6
Lei^9^	China	Cohort study	all-stage	ccRCC + non-ccRCC	75	31/34(≥50 y/<50 y)	C	≥25%	NA	OS	8
Weber^17^	Germany	Cohort study	localized	ccRCC	132	Median 63.5	C+N	≥10%	122.4	CSS	8
Liu^18^	China	Cohort study	all-stage	ccRCC	90	Mean 52.2	C	>0	48.7	PFS	8
Lu^19^	China	Cohort study	all-stage	ccRCC	98	Mean 55.2	C	≥10%	NA	OS	6
Shi^20^	China	Cohort study	all-stage	ccRCC	65	Mean 59.8	C	≥5%	19	OS	7

C: cytoplasm; N: nucleus; OS: overall survival; CSS: cancer-specific survival; PFS: progression-free survival; NA: not available.

^a^Reported at time of dianosis

^b^157 patients ≥65 years, and other 155 patients <65 years.

^c^IHC score was measured by computer assisted analysis with the IHCScore software.

^*^The quality of the included studies was evaluated using the Newcastle–Ottawa scale.

**Table 2:** Subgroup analysis of pooled HR for RCC patients with survivin overexpression.

Outcome	Subgroup	Studies	Pooled HR	95% CI	P Value	Model	Heterogeneity*I*^*2*^ (%)	P Value
OS	Ethnicity							
	Caucasian	0	–	–	–	–	–	–
	Asian	4	2.84	1.68–4.79	<0.001	fixed	6.9	0.359
	Subcellular location							
	nucleus	0	–	–	–	–	–	–
	cytoplasm	4	2.84	1.68–4.79	<0.001	fixed	6.9	0.359
	Extent of tumor							
	all-stage	4	2.84	1.68–4.79	<0.001	fixed	6.9	0.359
	localized	0	–	–	–	–	–	–
	metastatic	0	–	–	–	–	–	–
	Histopathological subtype							
	ccRCC	2	7.37	2.21–24.58	0.001	fixed	0	0.722
	Cutoff of staining							
	< 10%	1	5.98	1.13–31.67	0.036	–	–	–
	≥ 10%	3	2.61	1.51–4.54	0.001	fixed	15.7	0.306
CSS	Ethnicity							
	Caucasian	8	2.36	1.41–3.95	0.001	random	90.8	<0.001
	Asian	0	–	–	–	–	–	–
	Subcellular location							
	nucleus	5	2.28	1.20–4.35	0.012	random	94.5	<0.001
	cytoplasm	3	2.33	1.40–3.88	0.001	fixed	0	0.688
	Extent of tumor							
	all-stage	6	2.31	1.30–4.10	0.004	random	93.2	<0.001
	localized	2	2.75	1.23–6.15	0.014	fixed	0	0.919
	metastatic	0	–	–	–	–	–	–
	Histopathological subtype							
	ccRCC	8	2.36	1.41–3.95	0.001	random	90.8	<0.001
	Cutoff of staining							
	< 10%	2	1.95	1.35–2.83	<0.001	fixed	0	0.762
	≥ 10%	3	3.03	1.54–5.98	0.001	fixed	0	0.903
PFS	Ethnicity							
	Caucasian	2	1.96	1.28–3.01	0.002	fixed	0	0.913
	Asian	2	2.64	1.54–4.53	<0.001	fixed	0	0.838
	Subcellular location							
	nucleus	1	1.93	1.14–3.27	0.015	–	–	–
	cytoplasm	3	2.41	1.56–3.72	<0.001	fixed	0	0.833
	Extent of tumor							
	all-stage	4	2.20	1.58–3.08	<0.001	fixed	0	0.857
	localized	0	–	–	–	–	–	–
	metastatic	0	–	–	–	–	–	–
	Histopathological subtype							
	ccRCC	3	2.14	1.51–3.04	<0.001	fixed	0	0.782
	Cutoff of staining							
	< 10%	3	2.14	1.51–3.04	<0.001	fixed	0	<0.001
	≥ 10%	1	2.92	0.97–8.80	0.057	–	–	–

OS: overall survival; CSS: cancer-specific survival; PFS: progression-free survival; HR: hazard ratio; CI: confidence interval; RCC: renal cell carcinoma; ccRCC: clear cell renal cell carcinoma.

**Table 3:** Meta-analysis of the association between high survivin expression and clinicopathological features of RCC.

Variables	Studies	Pooled RR	95% CI	P Value	Model	Heterogeneity*I*^*2*^ (%)	P Value
TNM stage	5	2.75	2.21–3.44	<0.001	fixed	0	0.520
pT stage	5	2.19	1.75–2.75	<0.001	fixed	12.2	0.336
Lymph node metastasis	6	2.28	1.61–3.25	<0.001	fixed	0	0.514
Distant metastasis	4	1.56	1.16–2.08	0.003	random	85.7	<0.001
Fuhrman grade	6	2.81	2.29–3.45	<0.001	fixed	0	0.618
Tumor size	4	1.49	1.24–1.78	<0.001	random	91.9	<0.001

RR: relative ratio; CI: confidence interval; RCC: renal cell carcinoma.

**Table 4:** Pooled HR (95% CI) of sensitivity analysis for the effect of survivin expression on CSS.

Study Omitted	Pooled HR	95% CI	P Value	Model	Heterogeneity *I*^*2*^ (%)	P Value
Kosari^12^	2.42	1.36–4.32	0.003	random	92	<0.001
Parker^11^	2.47	1.36–4.50	0.003	random	92	<0.001
Krambeck^13^	2.42	1.35–4.33	0.003	random	92	<0.001
Zamparese^14^	2.26	1.32–3.88	0.003	random	91.9	<0.001
Parker^15^	1.39	1.27–1.53	<0.001	fixed	47	0.079
Eckel-Passow^16^	2.68	1.81–3.97	<0.001	random	61.3	0.017
Weber(Nucleus)^17^	2.32	1.33–4.03	0.003	random	91.9	<0.001
Weber(Cytoplasm)^17^	2.36	1.39–4.01	0.002	random	92.1	<0.001
Combined	2.36	1.41–3.95	0.001	random	90.8	<0.001

HR: hazard ratio; CI: confidence interval; CSS: cancer-specific survival.
